# The Regulatory Role of miRNAs in Zebrafish Fin Regeneration

**DOI:** 10.3390/ijms251910542

**Published:** 2024-09-30

**Authors:** Jiaqi Fan, Xinya Liu, Ziheng Duan, Hanya Zhao, Zhongjie Chang, Li Li

**Affiliations:** Molecular and Genetic Laboratory, College of Life Science, Henan Normal University, 46# East of Construction Road, Xinxiang 453007, China; f18236121381@163.com (J.F.);

**Keywords:** miRNAs, blastema, bone formation, osteoblast, fin

## Abstract

Since Teleostei fins have a strong regenerative capacity, further research was conducted on the regulation of gene expression during fin regeneration. This research focuses on miRNA, which is a key post-transcriptional regulatory molecule. In this study, a miRNA library for the fin regeneration of zebrafish was constructed to reveal the differential expression of miRNA during fin regeneration and to explore the regulatory pathway for fin regeneration. Following the injection of miRNA agomir into zebrafish, the proliferation of blastema cells and the overall fin regeneration area were significantly reduced. It was observed that the miRNAs impaired blastocyte formation by affecting fin regeneration through the inhibition of the expressions of genes and proteins associated with blastocyte formation (including *yap1* and Smad1/5/9), which is an effect associated with the Hippo pathway. Furthermore, it has been demonstrated that miRNAs can impair the patterns and mineralization of newly formed fin rays. The miRNAs influenced fin regeneration by inhibiting the expression of a range of bone-related genes and proteins in osteoblast lineages, including *sp7*, *runx2a*, and *runx2b.* This study provides a valuable reference for the further exploration of morphological bone reconstruction in aquatic vertebrates.

## 1. Introduction

The regenerative capacities of vertebrates differ by species, with lower vertebrates possessing stronger regenerative capabilities. In contrast to mammals, amphibians and fish can completely regenerate damaged or lost organs. Zebrafish (at adult, larval, or embryonic stages) is a model organism ideal for the study of the cellular and molecular events of organogenesis and regeneration due to their external development, rapid sexual maturation, optical transparency of larvae during embryogenesis, and ability to accelerate genetic research through gene knockout or overexpression, as well as possessing over 70% of the genes shared with humans [1,2,3,4,5]. They can regenerate multiple tissues and organs, including the heart, retina, scales, and fins [6,7,8,9]. Among zebrafish body structures, caudal fins are easily observable on the surface. With a rapid regeneration speed and simple structure, they serve as an excellent molecular system for the study of development and regeneration. Fins have an exoskeleton structure with regenerative abilities, which is covered with an epidermis on the surface while being internally composed of segmented, bony lepidotrichia and soft unmineralized actinotrichia [10]. The lepidotrichia comprise a pair of segmented hemirays, resembling a parenthesis. They protect blood vessels, nerve fibers, and connective tissue cells [10].

Subsequent to damage, the caudal fin completes regeneration through three stages of wound healing, blastema formation, and regrowth [11,12]. This involves multiple stages of cell dedifferentiation, differentiation, proliferation, migration, and the reconstruction of tissue structures [10].

Single-stranded miRNAs are non-coding RNA molecules comprising 19–24 nucleotides that are widely present in eukaryotes, which play essential roles in the regulation of gene expression [13]. The production of miRNAs involves a series of biological processes, with two important post-transcriptional processing steps being carried out by the DROSHA enzyme, located in the nucleus, and the DICER enzyme located in the cytoplasm [14,15]. Mature miRNAs are assembled with Argonaute (AGO) proteins to create an RNA-induced silencing complex (RISC), which involves the loading of double-stranded miRNAs and unwinding [16]. At this juncture, one strand of the double-stranded miRNAs in the RISC is selected as the mature miRNAs, while the passenger strand is released and quickly degraded. At this stage, mature miRNAs can regulate target genes [17]. The exact mechanism behind the regulation of gene expression by miRNAs through RISC is not fully understood. However, the complementarity between miRNAs and their target gene binding sites is critical. High complementarity translates to mRNA degradation, while low complementarity inhibits translation [18,19]. The characteristic of miRNAs that can regulate gene expression with low complementarity enables them to broadly influence gene expression. Typically, miRNAs bind to mRNAs through the “seed sequence” of miRNAs, which pairs with the 3′ untranslated region (3′ UTR) of the target mRNA and leads to the inhibition of the translation or degradation of the target mRNA. According to the different modes of action of miRNAs, they can be divided into two categories: one is synthesized and spliced within the cell and directly binds to the mRNA of the target gene to exert its effect; the other is miRNA transmitted between cells, which does not directly regulate the target gene in the synthesized cell after synthesis and splicing, but leaves the generated cell through exocytosis after being encapsulated by vesicles and exerts its effect after being transported to the target cell.

The first evidence of miRNAs regulating bone development emerged from studies into the expression of Col2a1 in DICER-knockout chondrocytes, showing that a lack of DICER in embryos results in severe bone defects, which indicated that miRNAs played a critical role in bone formation [20]. Bone development and remodeling involve multiple gene expressions, and where non-coding RNAs (ncRNAs) play an important role, with miRNAs regulating their targets particularly effectively [21]. All these results emphasize the critical role of miRNAs in bone development.

Regulators of bone formation include essential transcription factors, such as *runx2* and *sp7*, as well as major signaling pathways (e.g., FGF, WNT, BMP, and Hippo) [22,23,24,25]. For fin regeneration, osteoblasts must continuously differentiate to lengthen the fin rays. The key genes involved in this process include *runx2*, *sp7*, and *yap1*. For the regeneration of fish fins, *runx2* is one of the earliest expressed genes, where self-renewing bone progenitor cells express *runx2* in proximity to the distal blastema at an early stage. In contrast, osteoblasts express *runx2* and *sp7* near the proximal blastema. miR-25b regulates cell proliferation by targeting transcription factor *sp7* to control osteoblast differentiation [26]. miR-3658 targets *runx2*, reducing its expression, activating *runx2* expression, promoting osteoblast differentiation, and facilitating bone formation in zebrafish scale regeneration [27]. Studies have shown that *yap1* regulates the early stages of fin regeneration through the control of cell proliferation and migration [22,28]. miR-214 inhibits the osteogenic differentiation of hBMSCs by downregulating *yap1* [29].

For this study, five periods of 0 days post-amputation (dpa), 2 dpa, 4 dpa, 7 dpa, and 15 dpa were selected to construct an miRNA library and elucidate the regulatory role of miRNAs in the regeneration of caudal fins for zebrafish. The overexpression of miRNAs resulted in a decrease in the proliferation of blastema cells, diminished expression of genes related to germ formation, and impaired regenerative outgrowth. Furthermore, overexpressed miRNAs affected bone formation by inhibiting the differentiation of osteoblasts.

## 2. Results

### 2.1. Analysis of miRNA Differential Expression

**Figure 1:**
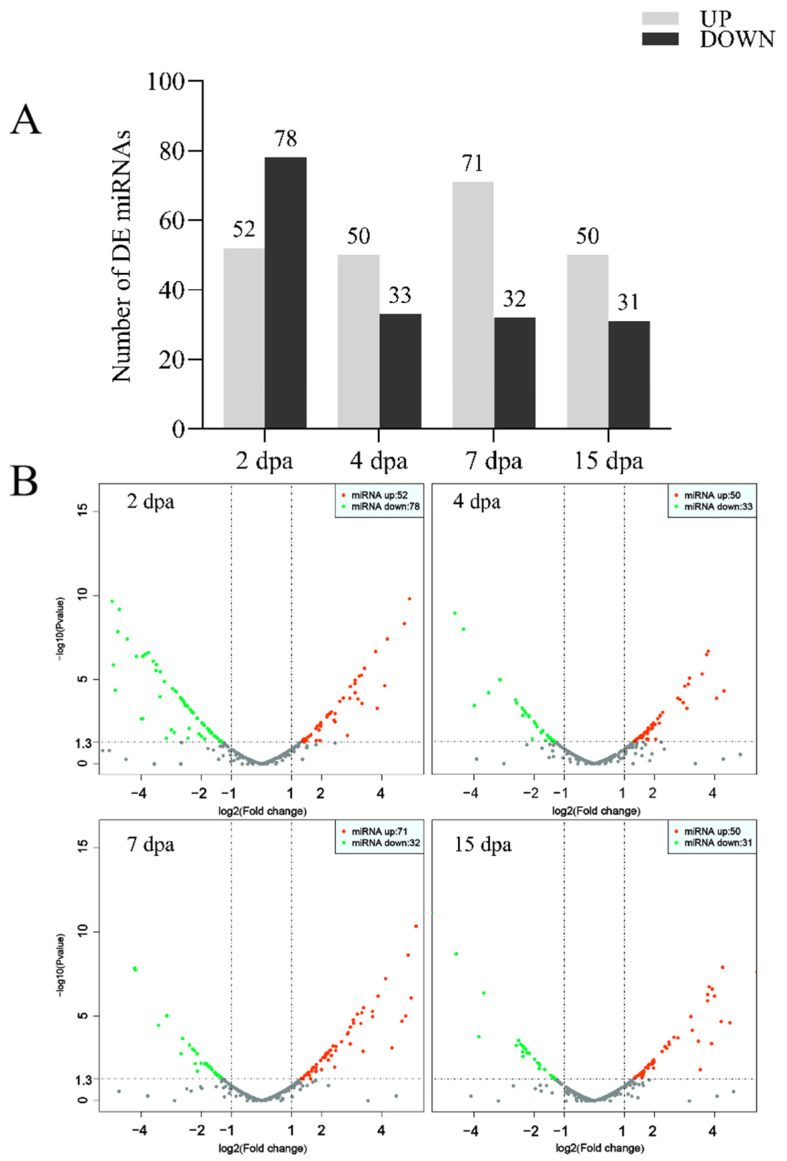

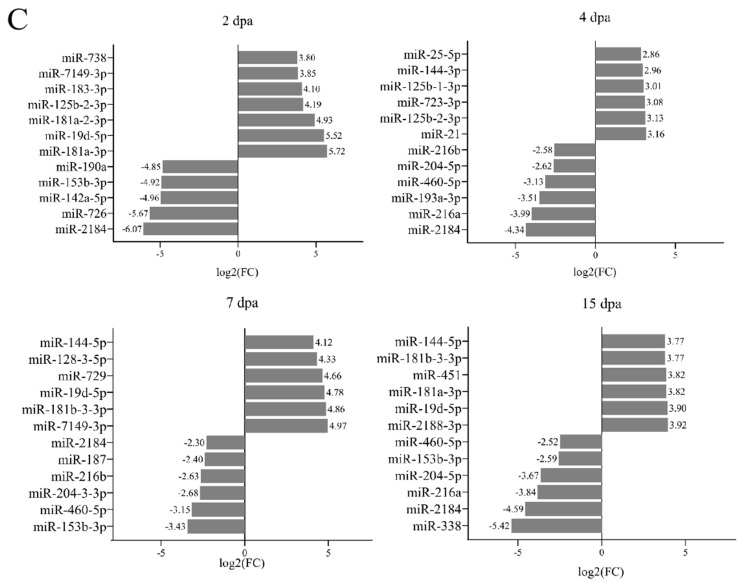
Differential expression analysis of miRNA in caudal fin regeneration. (**A**) Differential expression of miRNAs in the five fin regeneration stages. (**B**) Distribution of the differently expressed miRNAs. (**C**) The top 12 differential expression miRNAs.

Compared with the control group, 130 known miRNAs exhibited significant changes in expression in the group at 2 dpa (52 upregulated and 78 downregulated). Considerable differences in the expressions of 83 known miRNAs were observed in the 4 dpa group (50 upregulated and 33 downregulated). Substantial differential expressions of 103 known miRNAs were observed in the 7 dpa group (71 upregulated and 32 downregulated). Finally, 81 miRNAs revealed noteworthy modified expressions in the 15 dpa group (50 upregulated and 31 downregulated) (Figure 1A,B). These findings provide important evidence for further understanding the regulatory mechanisms of miRNAs at different developmental stages. There are 12 miRNAs in each group, including six upregulated miRNAs and six downregulated miRNAs, with the highest coefficient of variation (Figure 1C).

To elucidate the potential differentially expressed molecular functions of miRNAs at five different stages during zebrafish fin regeneration, miRanda (Ver. 3.3), pita, and RNAhybrid (Ver. 2.1.2) software programs were employed to predict miRNA target genes, with the intersection taken as the predictive result. Among these target genes, many were related to zebrafish fin regeneration (e.g., *sp7*, *runx2*, *sox9*, *yap1*, etc.). The results reveal many miRNA-targeted genes related to fish fin regeneration (e.g., miRNA-218a targeting *runx2a* and miRNA-145-5p targeting *runx2b*). Moreover, both miRNA-205 and miRNA-217 were predicted to target *sox9a*, while both miRNA-338 and miRNA-192 were predicted to target *sp7*, with each of them maintaining low expression levels throughout the zebrafish fin regeneration period. Furthermore, *yap1* was likely a target gene of miRNA-375, miRNA-124, and miRNA-144.

Following the KEGG pathway analysis of differentially expressed miRNA target genes, eight of 155 enriched pathways were found to be closely related to regenerative mechanisms, including Notch, WNT, MAPK, FOXO, Insulin, and Hippo signaling pathways. From the enrichment of genes regulated by differentially expressed miRNAs, it was clear that the vast majority of target genes were intimately related to the development, immunity, and metabolism of zebrafish. It is noteworthy that the analytical results of the target gene KEGG signaling pathway and GO annotation for differentially expressed miRNA are highly consistent with the corresponding mRNA sequencing analysis results for zebrafish fin regeneration conducted in previous studies (Figure S1).

### 2.2. In Vitro Validation of the Targeting Relationship between miRNA and Predicted Target Genes

**Figure 2:**
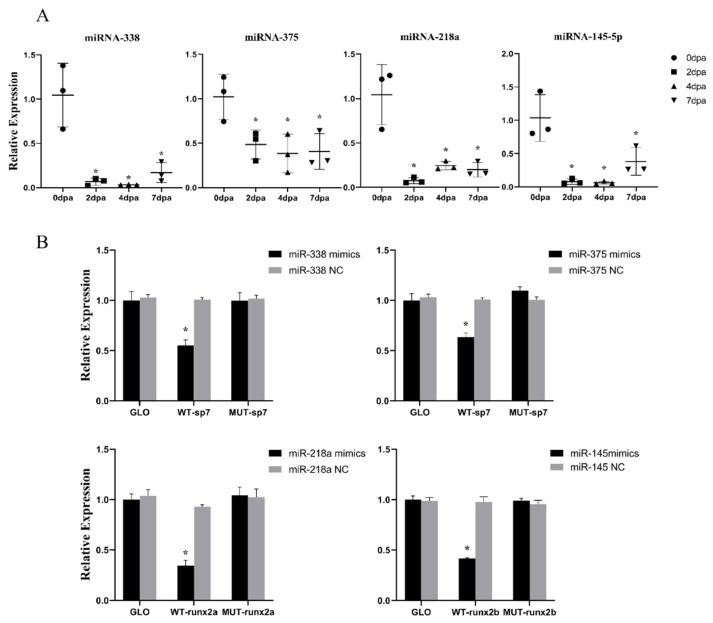
In vitro validation of the targeting relationship between miRNA and predicted target genes. (**A**) Differential expressions of miR-338, miR-375, miR-218a, and miR-145-5p in the four stages. (**B**) Luciferase assays of miR-mimics and miR-NC co-transfected with WT and MT plasmids in HEK293T cells. Significant differences (*p* < 0.05) between the treatment and control groups are indicated by asterisks above the bars.

It was found that miR-338, miR-375, miR-218a, and miR-145-5p maintained low expression levels throughout the fin regeneration period. Quantitative real-time PCR (RT-qPCR) showed that, in contrast to intact fins, the expressions of these four miRNAs in regenerated fins were significantly downregulated at 2 dpa, reaching their lowest point at 4 dpa. At 7 dpa, the expression levels of all four miRNAs increased, but remained significantly lower than those at 0 dpa (Figure 2A).

Dual-luciferase reporter assays were performed to detect whether these four miRNAs could regulate their target genes predicted from three authoritative databases: RNAhybrid, pita, and Targetfish. The results reveal that, in each group, dual-luciferase activity is significantly lower in the WT vector co-transfected with miRNA agomir than in the empty vector group (GLO), whereas the dual-luciferase activity of the mutant vector group (MUT) co-transfected with miRNA is not altered (Figure 2B).

### 2.3. Overexpression of miRNAs Affects Regenerative Outgrowth

**Figure 3:**
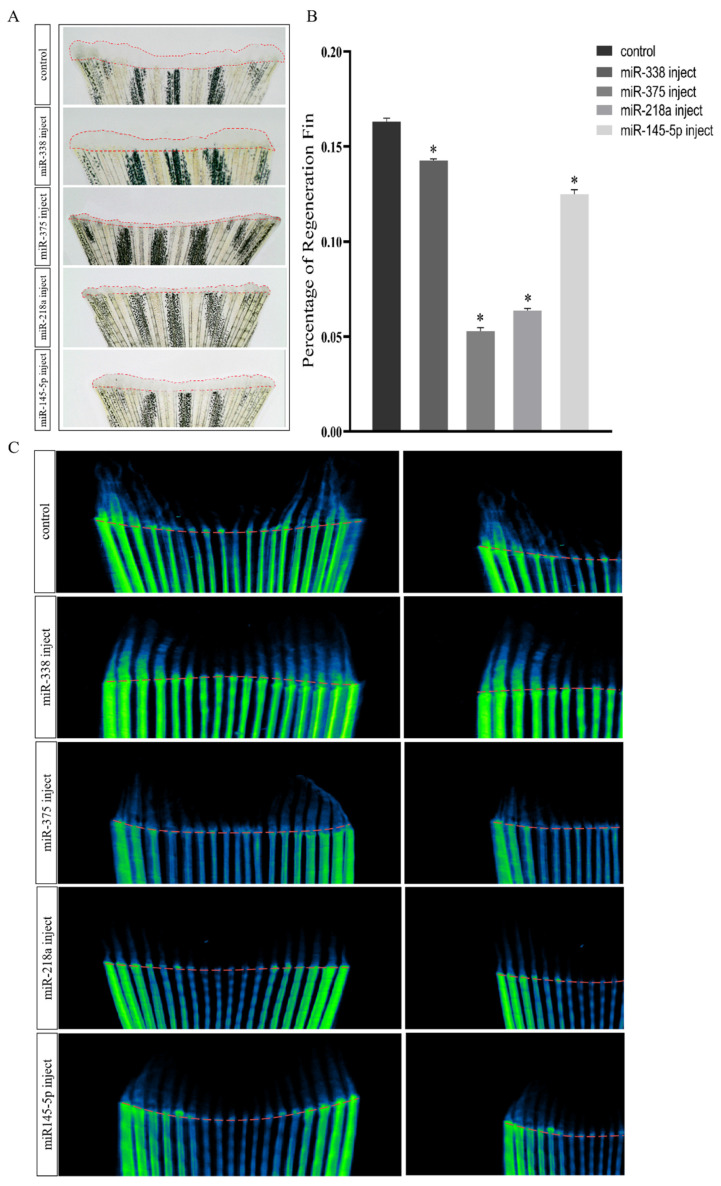
Analysis of phenotypic changes in caudal fin regeneration following miRNA injection. (**A**) Regenerative phenotype of the 4 dpa regeneration group and the miRNA injection group. (**B**) Percentage of the total caudal fin area of each group (*n* = 4 fish in each group). (**C**) CT presentation of 4 dpa regeneration group and miRNAs injection group. Significant differences (*p* < 0.05) between treatment and control groups are indicated by asterisks above the bars.

These four miRNA overexpression models were constructed by injecting specifically modified miRNA mimetics. On observing the growth of regenerated zebrafish fins in the overexpression model, it was found that, compared with the control group, the regeneration lengths of fins in the near far axis were significantly reduced in the miR-375 and miR-218a injection groups. Furthermore, the inhibition of fin regeneration was insignificant in the miR-338 and miR-145-5p injection groups. Following quantifications, including calculations of the percentages of fin regeneration related to the overall fin areas, no significant decreases in the regenerated fin areas were observed in the miR-338 injection group. The regenerated areas of the fins in all other groups were observed to be significantly reduced (Figure 3A,B). Micro-computed tomography (micro-CT) was also performed to further elucidate the regeneration of the fish fins. The results indicate that, compared with the control group, only the miR-338 injection group exhibits nearly half of the regenerated bone with a similar bone density to the original in the regenerated fins. The other groups showed only trace amounts of regenerated bone with a similar bone density to the original at the truncation sites (Figure 3C). The negative control group (miRNA-NC injected) did not show significant changes compared to the blank control group (Figure S3).

### 2.4. miRNAs Regulate Fish Fin Regeneration by Inhibiting Blastema Formation

**Figure 4:**
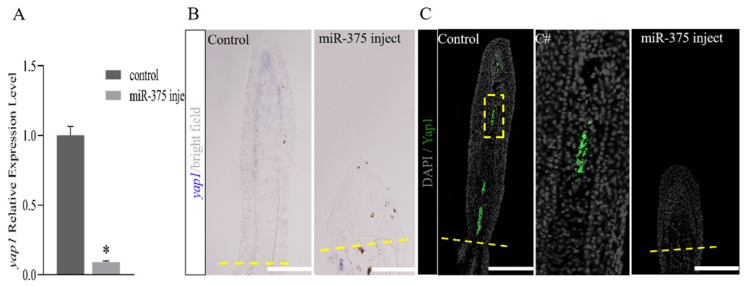

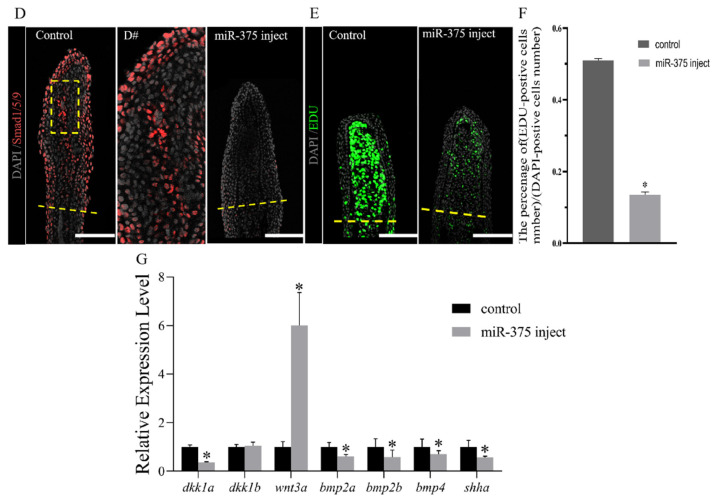
miRNA regulates fin regeneration by inhibiting the Hippo-Yap pathway. (**A**) Quantitative real-time PCR analysis of *yap1* mRNA of the caudal fin. (**B**) In situ hybridization on cryosections at 4 dpa (1 dpi) illustrating a severe reduction in *yap1* expression located at the distal blastema under miRNA-mimics injection in contrast to the control group. (**C**) Immunodetection of osteoblasts using the Yap1 osteoblast-specific antibody on longitudinal sections of fins regenerates at 4 dpa (1 dpi) after miR-375 injection and control. In the control rays, more basal cells are found on the surfaces of the distal lepidotrichia. (C#) Magnification of yellow dotted box in C. (**D**) Immunodetection of osteoblasts using the Smad1/5/9 osteoblast-specific antibody on longitudinal sections of regenerated fins at 4 dpa (1 dpi) after miR-375 injection and control. In the control rays, more basal cells are found on the surfaces of the distal lepidotrichia. (D#) Magnification of yellow dotted box in D. (**E**) Fluorescent detection of DNA-replicating cells in regenerated fin at 4 dpa, 12 h following 5-Ethynyl-20-deoxyuridine (EdU) injection and miR-375 injection, and the control at 1 dpa. (**F**) Statistical analysis of EdU-positive cells in sections of fin rays (*n* = 4 rays and 6 sections). (**G**) Analysis of the relative expression changes in upstream and downstream *yap1*-related genes during miR-375 injection. Dashed lines indicate the amputation plane. Plot values represent mean ± s.d. Significant differences (*p* < 0.05) between treatment and control groups are indicated by asterisks above the bars. Scale bar: 100 μm (**B**–**F**).

Subsequent to the amputation of the caudal fin of a zebrafish, epithelial cells migrated and covered the surface of the wound to form an apical epidermal cap (AEC). Beneath the AEC, abundant undifferentiated mesenchymal cells gathered to form a blastema, which was critical for the completion of the regenerative process [10]. As an important downstream effector of the Hippo pathway, *yap1* played important roles in the development of the blastema and initiation of outgrowth [30]. RT-qPCR was performed on the expression of *yap1* in the control and miR-375 injection groups. It was found that *yap1* expression was significantly downregulated in the miR-375 injection group (Figure 4A). To detect changes in the nucleic acid levels, in situ hybridization (ISH) was performed, with the results showing that the *yap1* signal in the control group is primarily concentrated in the distal blastema of regenerated fish fins, with a small amount expressed along the lepidotrichia. The expression level in the proximal end of the lepidotrichia was significantly lower than in the distal end. Following miR-375 injection, the expression of *yap1* at the distal end disappeared (Figure 4B). Furthermore, compared with the control group, immunostaining using the Yap1 antibody showed almost no Yap1 expression in the miR-375 injection group (Figure 4C). As reported in the previous research, *yap1* regulates the BMP signaling pathway, while Smad1 and Smad5 are the pivotal intracellular effectors of the bone morphogenetic protein (BMP) family [22,31]. Compared with the control group, the number of Smad1/5/9-positive cells located in the distal blastema cells in the miRNA injection group was significantly reduced (Figure 4D). Cell proliferation was assessed by labeling DNA-replicating cells with 5-Ethynyl-20-deoxyuridine (EdU) for 12 h following miR-375 injection, and the collection of fins at 4 dpa. The miR-375 injection group exhibited a significant decrease in proliferative cells labeled with EdU in the blastema, in contrast to the control group (Figure 4E,F). As Yap1 is a downstream effector of the Hippo signaling pathway, its activation can trigger a phosphorylation cascade reaction, regulate the expression of numerous genes, and control multiple regeneration processes. For this reason, early regeneration marker genes were identified. The results reveal that, compared with the control group, the expression of *dkk1b* remains unchanged in the miR-375 injection group, while that of *wnt3a* is upregulated, with *dkk1a*, *bmp2*, *bmp4*, and *shha* being significantly downregulated (Figure 4G).

### 2.5. miRNAs Regulate the Regeneration of Fish Fins by Delaying Bone Formation and Osteoblast Differentiation or Functionality

**Figure 5:**
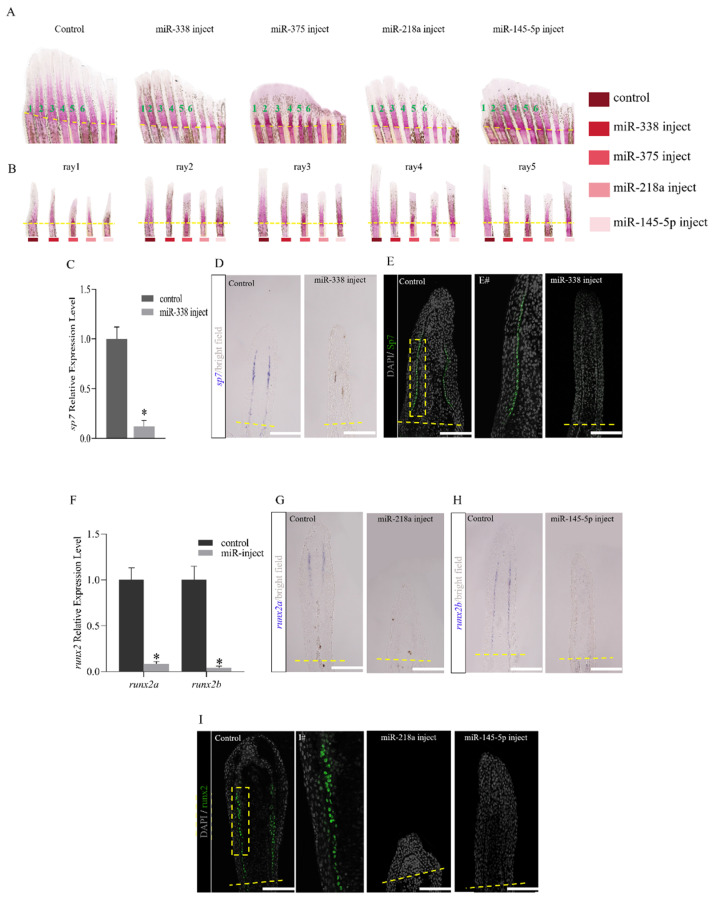
miRNAs regulate fin regeneration by inhibiting osteoblast differentiation. (**A**,**B**) Morphology of fin rays following miR-mimic injection. Analysis of mineralization progression using Alizarin red staining shows a reduction in matrix mineralization in rays 3–8 of fish injected with miRNA mimics at 8 dpa compared with control rays. Dashed lines indicate the amputation plane. (**C**) Quantitative real-time PCR analysis of *sp7* mRNA of the caudal fin. (**D**) In situ hybridization on cryosections at 4 dpa (1 dpi) illustrating a severe reduction in *sp7* expression along the distal-proximal axis in miRNA-mimic-injected groups compared to control groups. (**E**) Immunodetection of osteoblasts using Sp7 osteoblast-specific antibody on longitudinal sections of regenerated fins at 4 dpa (1 dpi) after miR-338 injection of control. In the control rays, more mature bone-secreting cells are found on the surfaces of the proximal lepidotrichia. (E#) Magnification of yellow dotted box in E. (**F**) Quantitative real-time PCR analysis of *runx2a* and *runx2b* mRNAs of caudal fin. (**G**) In situ hybridization on cryosections at 4 dpa (1 dpi) illustrating a severe reduction in *runx2a* expression along the distal–proximal axis in the miRNA-218a injected group compared to the control group. (**H**) In situ hybridization on cryosections at 4 dpa (1 dpi) illustrating a severe reduction in *runx2b* expression along the distal–proximal axis in miRNA-145-5p injected group compared to the control group. (**I**) Immunodetection of osteoblasts using the Runx2 osteoblast-specific antibody on longitudinal sections of regenerated fins at 4 dpa (1 dpi) after miR mimic injection and control. In the control rays, more mature bone-secreting cells are found on the surfaces of the proximal lepidotrichia. (I#) Magnification of yellow dotted box in (**I**). Dashed lines indicate the amputation plane. Plot values represent mean ± s.d. Significant differences (*p* < 0.05) between treatment and control groups are indicated by asterisks above the bars. Scale bar: 100 μm (**D**,**E**,**G**–**I**).

Staining with Alizarin red at 8 dpa following injection with miRNA mimics showed that the overexpression of miRNAs resulted in a reduction in bone matrix mineralization in all the examined rays (1–5), with a most noticeable effect on the five outermost rays (Figure 5A,B). A specific gene (*sp7*) is responsible for the differentiation of osteoblasts during bone formation. Quantitative analysis was conducted on *sp7* in the control group and the miR-338 injection group, which revealed that it was significantly downregulated in the injection group. The ISH results show that, for regenerated fish fins at 4 dpa, *sp7* is strongly activated along the distal–proximal axis in the control group. In contrast, the expression was severely reduced in miR-338 mimic injected fins (Figure 5C,D). For further validation, immunofluorescence histochemical detection was performed on the expression of Sp7 in regenerated fish fins. The results reveal that the Sp7 signal is expressed along the lepidotrichia in the control group, and there is a higher signal at the proximal end of the fin. As the fin extended toward the distal end, the expression gradually decreased. When miR-338 was overexpressed, the expression of Sp7 was significantly reduced (Figure 5E). Osteoblasts originate from osteoprogenitor cells that differentiate by gradually expressing mature markers, while immature cells express *runx2*. The results show that, compared with the control group, the expressions of *runx2a* and *runx2b* are significantly downregulated following injection with miR-218a and miR-145-5p, respectively (Figure 5F). Subsequently, in situ hybridization analysis was performed on *runx2a* and *runx2b*. The labeled sites of *runx2a* and *runx2b* were consistent with the osteoblast distribution sites. The expressions of *runx2a* and *runx2b* were very obvious in the control group, while the positive signals of *runx2a* and *runx2b* almost disappeared in the miR-218a and miR-145-5p injection groups (Figure 5G,H). Immunostaining indicated that, compared to the 4 dpa control group, the Runx2-positive signal disappeared in the miRNA injection groups (Figure 5I).

## 3. Discussion

Since the discovery of the first miRNAs in nematodes in 1993, an increasing number of miRNAs have been discovered in animals [32]. It is well known that miRNAs are involved in the regulation of developmental timing, cell proliferation, cell cycles, maintaining pluripotency and cell reprogramming, cell differentiation and cell fate, regulation of gene expression networks, fine-tuning of gene expression, and maintenance of phenotypic robustness [33]. Most often, miRNAs bind to specific sites in the 3′ UTR of target mRNAs to regulate them. They can induce the cleavage or decay of target genes by interfering with the stability of mRNAs or preventing binding between ribosomes and mRNAs [34,35,36]. In fact, miRNAs that are expressed at lower levels in the process of regeneration tend to regulate genes required for regeneration, while those that are expressed at increased levels typically have anti-proliferative effects or are involved in maintaining cellular fate [37]. miR-133 was highly expressed in normal fish fins and played an important role in maintaining the growth of fish fins [38]. Thatcher used gene chips to detect the expression changes in miRNAs during zebrafish fin regeneration, which emphasized the importance of a complete miRNA pathway [37]. Subsequently, there are few reports on the research into miRNAs in zebrafish fin regeneration over extended periods. This study selected five periods to construct a miRNA library: a control group at 0 dpa; pre-regeneration at 2 and 4 dpa, with a high expression of regeneration-related genes; mid-regeneration at 7 dpa, when genes resume expression and enter the growth stage; and late-regeneration at 15 dpa, when regeneration is basically completed with some genes still not recovered. These periods are critical for fin regeneration, where the study of changes in miRNA expression is critical for understanding the regulatory mechanisms. After RT-qPCR validation, the results of RT-qPCR for 12 randomly selected miRNAs are consistent with the sequencing results, which confirm the high quality and reliability of the constructed library. Sequencing revealed that certain miRNAs maintained a high expression under normal conditions and during regeneration, which suggested that they may be involved in maintaining basic cellular activities. Differentially expressed miRNAs during regeneration may play an important regulatory role in cell differentiation. Research has found that, compared with the control group, numerous miRNAs show differential expressions during regeneration, with some maintaining a low expression throughout the regeneration period, which was speculated to be associated with the initiation of regeneration. KEGG pathway analysis was conducted on the target genes of differentially expressed miRNAs, with the results showing that 156 signaling pathways are involved. Among them, at least eight pathways were closely related to the regeneration and development of zebrafish. These signaling pathways play crucial roles in tissue regeneration and organ development, with some even directly participating in the regulation of regeneration. For example, the WNT signaling pathway is essential for the formation of blastemas during zebrafish fin regeneration [39]. The Hippo signaling pathway plays an important role in the proliferation of blastema cells and the control of the growth pattern of zebrafish fins during fish fin regeneration [22]. GO enrichment results show that the biological processes regulated by differentially expressed miRNAs are primarily enriched in three categories (cellular components, biological processes, and molecular functions). The analyses of the pathway and biological processes above indicated the potential roles of differentially expressed miRNAs in zebrafish fin regeneration.

miRNAs induce mRNA degradation or inhibit translation by binding to the 3′-UTR of target genes. In this study, it was speculated that miRNAs targeting key regeneration genes may be downregulated during regeneration to promote target gene expression. After analyzing the differential expressions of miRNAs at five different time points, it was found that significantly downregulated miRNAs during regeneration were mainly concentrated at 2 dpa and 4 dpa. Among these miRNAs, miRNA-338, miRNA-375, miRNA-218a, and miRNA-145-5p consistently maintained low expression levels. Further predictions of their target genes combined with sequencing data showed that they targeted key genes related to regeneration. It was speculated that *runx2*, *sp7*, and *yap1* were target genes of miRNAs that were differentially expressed. *runx2* is involved in bone development, *sp7* is a transcription factor specifically expressed by osteoblasts, and *yap1* is related to the production and maintenance of germ cells and the normal growth of fins, all closely related to fin regeneration. In this study, decreased dual-luciferase activity once again indicated the recognition of the target gene ‘3’—UTR by these four miRNAs.

Fins are one of the most prominent features of a zebrafish’s body surface, which comprises bony lepidotrichia and unmineralized actinotrichia. The extension of the fin rays is achieved by increasing the number of bony segments toward the distal end. During the regenerative growth stage, mature and differentiated osteoblasts begin to appear in the outer region of the proliferating blastemas at the distal end, with this process occurring successively along the proximal–distal axis. The distal region includes pre-osteoblasts, while the proximal region includes osteoblasts that form bone and already differentiated osteoblasts, all of which participate in the formation of the two different types of fin rays to ultimately form a new fin that is identical to the one that was truncated.

Dicer is an important component of miRNA biosynthesis and is crucial for normal bone development. The research results indicate that the Dicer-dependent pathway plays a crucial role in regulating chondrocyte proliferation and differentiation during skeletal development [20]. Transgenic mice expressing miR-206 in osteoblasts developed a low bone mass phenotype due to impaired osteoblast differentiation [40]. BMP2 controls bone cell determination by inducing miRNAs that target muscle genes [41] All of these indicate that miRNAs can control the complex processes of osteogenic differentiation and bone formation in fish fins. miR-203 can target *lef1*, thereby inhibiting regeneration, while its absence can lead to the excessive expression of *lef1*, causing fish fin overgrowth [37]. A high expression of miR-133 can maintain the proper growth of fish fins and regulate Mps1 kinase, thereby guiding the proliferation of blastema cells and tissue renewal [38,42]. The complete miRNA regulatory pathway plays an important role in zebrafish fin regeneration. Through the construction of an overexpression model, the injection of miRNAs was found to significantly inhibit the regeneration of fish fins, with a significantly lower regeneration area than the control group, which indicated that they likely regulate regeneration. Although no significant inhibition was observed in the miR-338 injection group, the downregulated expression of *sp7* remained significant, indicating potential regulation. Furthermore, the micro-CT results once again confirm our hypothesis. Subsequently, in-depth research was conducted on the changes in target genes corresponding to these four overexpression models.

Hippo signaling is a conserved signaling pathway that, upon activation, leads to phosphorylation, cytoplasmic isolation, and the degradation of its effector YES-related protein 1 (YAP, also referred to as YAP1) and transcription co-activator with a PDZ binding motif (TAZ, also known as WWTR1). When inactivated, YAP and TAZ are transferred to the nucleus and regulate target gene expression [43,44]. This pathway plays a critical role in tissue growth and regeneration through the regulation of cell proliferation, survival, and fate determination [45,46]. In our previous results, sequencing and bioinformatics analysis of the transcriptome of fin regeneration showed the potential regulation of the Hippo signaling pathway in fin regeneration. In our more recent research, it was found that the *yap1* gene was significantly upregulated at 4 dpa during fin regeneration, as was shown by the ISH and immunofluorescence results. As previously mentioned, following miR-375 injection, the regeneration of fish fins was significantly inhibited. The results of the dual-luciferase reporter gene assay confirm our prediction of the miR-375 regulation of *yap1* 3′-UTR. Combined with the RT-qPCR results of *yap1* in the miR-375 injection group, it was speculated that miR-375 injection inhibited *yap1* and thereby affected the regeneration process. ISH and immunofluorescence results show that the *yap1* signal in the mRNA and protein disappears in the miR-375 injection group compared with the control group, indicating that miR-375 has inhibitory effects on both nucleic acid and protein levels during regeneration. As previously reported, the Hippo pathway effector Yap1 is associated with multiple signaling pathways, such as BMP and WNT. Research has indicated that *yap1* in mesenchymal cells can induce the activation of the BMP signaling pathway. BMP is active in the distal blastema, wound epidermis, and osteoblasts [22]. Furthermore, the normal expression of its pathway is correlated with the proliferation of blastema cells [47]. Immunofluorescence results indicate that, in the miR-375 injection group, Smad1/5 expression in distal blastema cells disappears. Followed by EdU detection, this showed that the number of distal blastema cells with proliferation significantly decreased following miR-375 injection. *dkk1a* and *dkk1b* are two DKK protein genes with similar expression patterns that are inhibitors of the WNT/β-catenin signaling pathway. In addition, BMP signaling promotes Dkk1b secretions to restrict WNT signaling to maintain progenitor cells in DB. Transient *yap1* inhibition leads to a significant decrease in *dkk1a* expression during fin regeneration, which indicates that *yap1* affects WNT signaling during fin regeneration [22]. In this study, miR-375 injection translated to a significant downregulation of *dkk1a* and a substantial increase in *wnt3a* expression. This indicated that the BMP signaling pathway was inhibited. Meanwhile, other genes involved in blastema formation also underwent downregulated expression. Therefore, it was speculated that miR-375 could regulate fin regeneration by modulating the formation of blastema.

As described earlier, the targeting relationships between miR-338 to *sp7*, miR-218a to *runx2a*, as well as miR-145-5p to *runx2b* were verified at the cellular level. Experiments with Alizarin red revealed that bone regeneration was inhibited in the miRNA injection groups, to varying degrees. Runx2 homozygous defective mice die soon after birth due to respiratory failure, with impaired mesenchymal osteoblast development, which results in the absence of osteocytes and bones. Heterozygous mice exhibit cleft palate hypoplasia, indicating that Runx2 is critical for bone development and formation. Sp7 is expressed in osteoblasts, and when defective, mice fail to form bones normally [24]. The knockdown of the *sp7* gene in Oryzias latipes results in severe skeletal developmental abnormalities and high mortality in juvenile fish [48]. The knockout of the *sp7* gene in zebrafish leads to reduced bone mineralization and abnormal tooth development in juvenile fish [49]. Thus, *sp7* is an excellent marker for the study of osteoblast differentiation. Interestingly, in this study, no significant regeneration inhibition was observed in fish fins injected with miR-338. However, ISH results imply that the expressions of *sp7*, *runx2a*, and *runx2b* are significantly reduced in the miRNA injection groups, with subsequent immunodetection revealing the disappearance of their protein signals. It was speculated that there was a compensatory mechanism that replaced the normal expression of *sp7* to perform its functions. It is known that *sp7* is a downstream target of *runx2,* which induces the expression of *sp7* [24,25,50]. Reports indicate that, in Sp7-deficient mice, Runx2+ pre-osteoblasts retain their ability to differentiate into osteoblasts and chondrocytes. Sp7 and WNT signaling inhibits chondrocyte differentiation and guides Runx2+ pre-osteoblasts to generate osteoblasts [51]. The regeneration of zebrafish fin bones is achieved through the dedifferentiation and proliferation of spared osteoblasts. Osteogenesis can occur even without *sp7*, indicating that there is a non-osteoblastic population that can differentiate into osteoblasts and drive fish fin regeneration. Singh et al. demonstrate diversity in the cellular origins of appendage bone and reveal that de novo osteoblasts can fully support the regeneration of amputated zebrafish fins [52]. Therefore, it was speculated that *sp7* and *runx2* may have an antagonistic effect. Due to the reverse regulatory effects of *sp7* on *runx2* that mediate the differentiation of pre-osteoblasts into osteoblasts, the expression of *sp7* is blocked during miR-338 injection. However, due to the low level of *sp7* expression, the inhibition of *runx2* is relieved, resulting in the high expression of *runx2*. At this time, the high expression of *runx2* continuously drives the differentiation and maturation of osteoblasts, which ultimately resulted in the negligible inhibition of fish fin regeneration in our zebrafish models. Relative quantitative analysis was performed on RT-qPCR of upstream and downstream genes from *sp7* during miR-338 injection, particularly for *runx2a* and *runx2b*. It was found that *runx2a* was significantly upregulated, while the in situ results also indirectly validate our speculation (Figure S2). Consequently, it was hypothesized that miRNAs might regulate fin regeneration through the modulation of bone formation.

## 4. Materials and Methods

### 4.1. Fish Care and Fin Amputations

The zebrafish (AB strain) used in this experiment were purchased from the National Zebrafish Resource Center and raised in an aquaculture system at a constant temperature (28.5 °C). To reduce experimental data errors, the zebrafish used in this experiment were all 6-month-old adults. Tricaine (0.02 mg/mL) was used to anesthetize the zebrafish during sampling.

### 4.2. Total RNA Isolation and miRNA-Seq

The total RNA was extracted from zebrafish fins at different days post-amputation (dpa) using the TRIzol reagent. Each regeneration time group was set with three replicates. In each repeat within each regeneration time group, 50 zebrafish fins were harvested. The extracted total RNA was mixed in the corresponding time group to form a total RNA pool, diluted to 200 ng/μL. The same method was used for each group to prepare the total RNA pool. The quality of the total extracted RNA pool was detected using the NanoDrop system and gel electrophoresis. Following qualification, it was used for machine sequencing (IlluminaHSeq™ 2500 sequencing).

### 4.3. Quantitative Real-Time PCR

cDNA was synthesized using the Takara PrimeScript™ RT Master Mix (Perfect Real Time). The first strand of miRNAs was synthesized using the Tiangen miRcut enhanced miRNAs cDNA first-strand synthesis kit (KR211). The RT-qPCR of the mRNAs was determined using the UltraSYBR Mixture (CW0957H, Cwbio, Taizhou, China). The RT-qPCR of the miRNAs was determined using the Tiangen miRcut enhanced miRNAs fluorescence quantitative detection kit (FP411). Elongation factor 1 alpha (*elfa*) gene and Danio rerio U6 snRNA biogenesis 1 (*usb1*) were employed as reference genes to normalize cDNA loading (Table S1).

### 4.4. Dual-Luciferase Reporter Gene Assay

According to RNAhybrid, pita, and Targetfish predictions, target gene 3′-UTR sequences containing miR-338, miR-375, miR-218a, and miR-145-5p binding sites were cloned from 4 dpa regenerated caudal fin cDNA using the PCR method. The cloned sequences were then integrated into a dual-luciferase reporter vector (WT Vector). Plasmids were extracted using the Plasma Mini Kit I (OMEGA) after being transformed into JM109 competent cells and cultured for 12 h. The mutation vector was synthesized via a chemical method (GenScript). Compared with the WT vector, the mutation vector was validated by sequencing, with only the miRNA binding site being different. The detection of dual-luciferase activities proceeded using the Dual-Luciferase Reporter AssaSystem Kit (Promega).

### 4.5. Drug Injections

Adult zebrafish of a consistent size were selected and anesthetized with tricaine. Subsequently, their caudal fins were removed from two sections after their first bifurcation point. At 1 dpa, intraperitoneal injections were administered, after which the caudal fins were collected at 4 dpa and 8 dpa for subsequent experiments. The dosage and injection method are summarized as follows: 0.68% sterile and enzyme-free physiological saline was used to dilute miRNA agomir to a concentration of 20 pmol for 15 μL injections per zebrafish. A pause of 30 s was observed following the injections prior to reintroducing the fish into the water. A total of 12 replicates were established for each model. A negative control group was established with the same number of replicates and injected with the same volume of physiological saline.

### 4.6. EdU (5-Ethynyl-20–Deoxyuridine) Labeling

The manufacturer’s instructions were followed during the EdU incorporation experiments. Fish were injected with 10 μL of 5 mg/mL EdU solution in sterile saline at 4 d after caudal fin amputation. After 12 h, the regenerated fins were harvested and the EdU was found using the Click-iT EdU Imaging Kit (C10337, Invitrogen, Waltham, MA, USA). Positive cells were enumerated using ImageJ’s (Ver. 1.54g) Threshold, Binary, and Analyze Particles.

### 4.7. In Situ Hybridization

In situ hybridization (ISH) on longitudinal cryosections of the caudal fins was performed, as previously described [53]. The *sp7*, *yap1*, *runx2a,* and *runx2b* cDNAs were cloned from mixed libraries of 4 dpa regenerated fins. The in situ hybridization primers were designed using the Primer 3 Input (Ver. 0.4.0) and Primer designing tool (NCBI primer blast) (Table S2). The *runx2a* probes were prepared as previously described [54].

### 4.8. Immunostaining

At 4 dpa, the fins were fixed in 4% PFA in PBS, embedded in OCT, and cryosectioned. Antibody staining was performed, as previously described [55]. The following primary antibodies were used: rabbit anti-Sp7 (ab209484, Abcam, Cambridge, UK), rabbit anti-Yap1 (ab52771, Abcam), rabbit anti-Runx2 (ab192256, Abcam), and rabbit anti-Smad1/5/9 (ab92698, Abcam). The following secondary antibodies were used: goat anti-rabbit Alexa Fluor 488 (ab150077, Abcam) and goat anti-rabbit Alexa Fluor 647 (ab1500779, Abcam).

### 4.9. Bone Staining and CT

At 8 dpa, the regenerated fins were fixed with 4% PFA at 4 °C overnight and stained by the “Two-color acid-free” method, as previously described [56]. The fish that had been regenerating their fins for 8 d were anesthetized and then euthanized on ice. They were then fixed overnight with 4% PFA and scanned using a micro-CT scanner (TY2021004774, BRUKER, Billerica, MA, USA).

### 4.10. Data Analysis

All data obtained in this experiment were derived from three parallel experiments, and the experimental materials were obtained from the same batch of purebred fish. The data were checked for normality and homogeneity of variance prior to conducting statistical comparisons, where error bars represented standard deviations. The differences between the treatments were determined by Duncan’s new multiple range test, where probability levels of *p* < 0.05 were considered statistically significant. Images for the experimental results were captured and analyzed by the official software of the Leica fluorescence confocal microscope (Ver. 4.0.0) and the Olympus BX51 microscope image acquisition system (Ver. 3.2).

## 5. Conclusions

Overall, this research focuses on the regulatory roles of miRNAs in the regeneration of zebrafish caudal fins. In this study, miRNAs played a critical role in zebrafish fin regeneration, as they prevented the regeneration of fins by inhibiting the formation of blastemas. Additionally, miRNAs impaired the patterns of newly formed fin lines and bone matrix deposition in zebrafish, which was possibly due to the inhibition of the expression of bone development marker genes and proteins in the osteoblast lineage. Thus, our research results provide valuable biological and additional reference data to facilitate an exploration of the regulation of miRNAs in zebrafish fin regeneration.

## Data Availability

All materials and analyzed data are included in this published article and the Supplementary Materials files.

## Supplementary Materials

The following supporting information can be downloaded at: https://www.mdpi.com/article/10.3390/ijms251910542/s1.
