# Supplementary material for: The Regulatory Role of miRNAs in Zebrafish Fin Regeneration
Source: Int J Mol Sci. 2024 Sep 30;25(19):10542. doi: 10.3390/ijms251910542 (PMC11477159; doi:10.3390/ijms251910542)
Supplement: Supplementary file 1 [file ijms-25-10542-s001.zip › Supplementary Figures S1-S3.pdf]

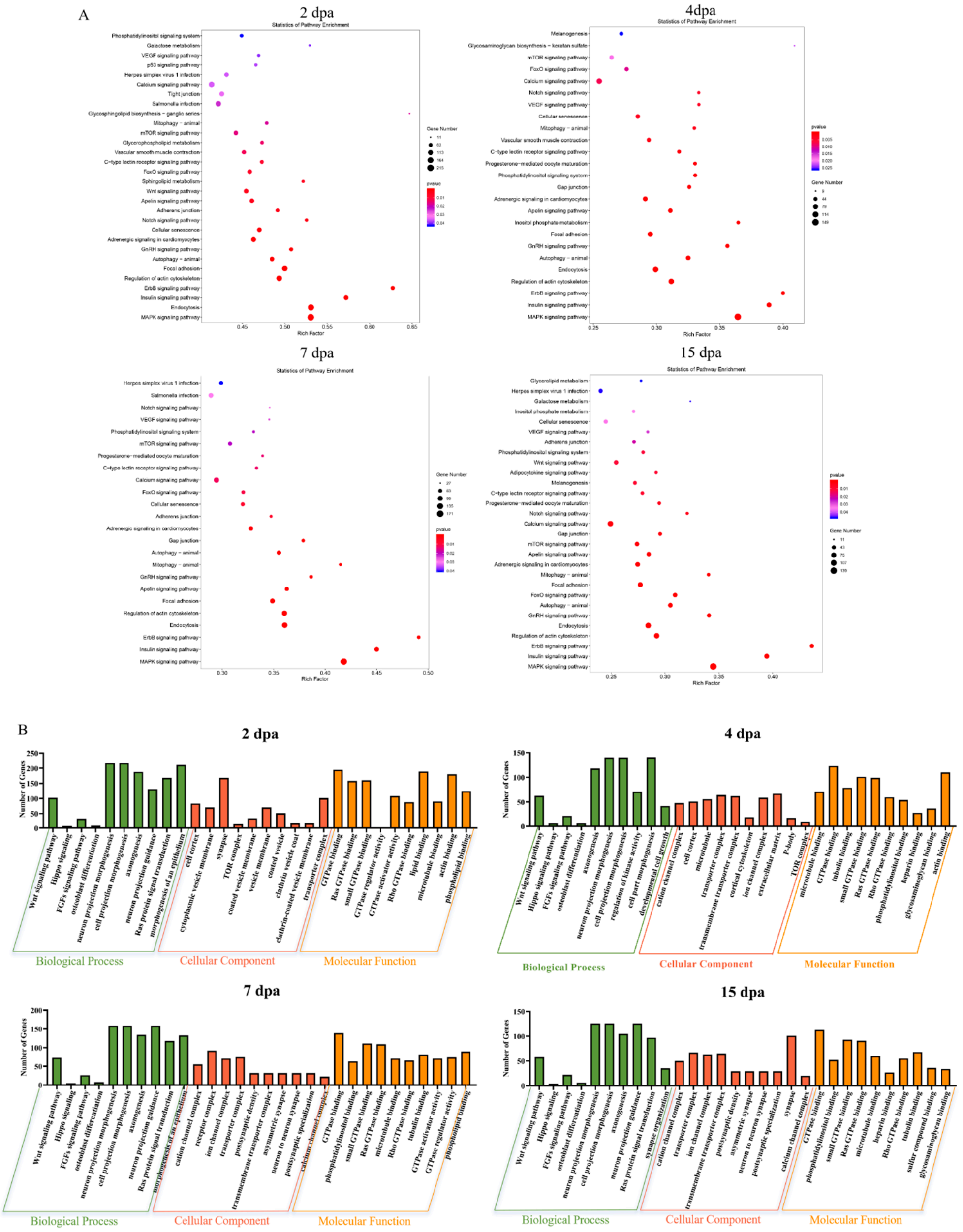

Figure S1. KEGG signaling pathway and GO annotation analysis of the predicted target genes. (**A**) KEGG enrichment analysis of predicted target genes by differentially expressed miRNAs at different regeneration times. (**B**) GO enrichment analysis of predicted target genes predicted by differentially expressed miRNAs at different regeneration times.

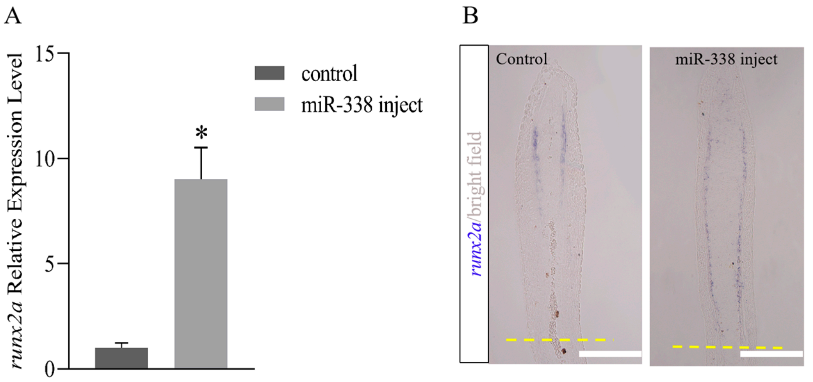

Figure S2. Expression of runx2a after injection of miR-338. (**A**) Quantitative real time PCR analysis of *runx2a* mRNA of caudal fin. (**B**) In situ hybridization on cryosections at 4 dpa (1 dpi) illustrating a severe reduction of r*unx2a* expression along the distal-proximal axis in miRNA-338 injected compared to control. Dashed lines indicate the amputation plane. Plot values represent means ±s.d. Significant differences ($p < 0.05$) between treatment and control groups are indicated by asterisks above the bars. Scale bars: 100 µm (**B**).

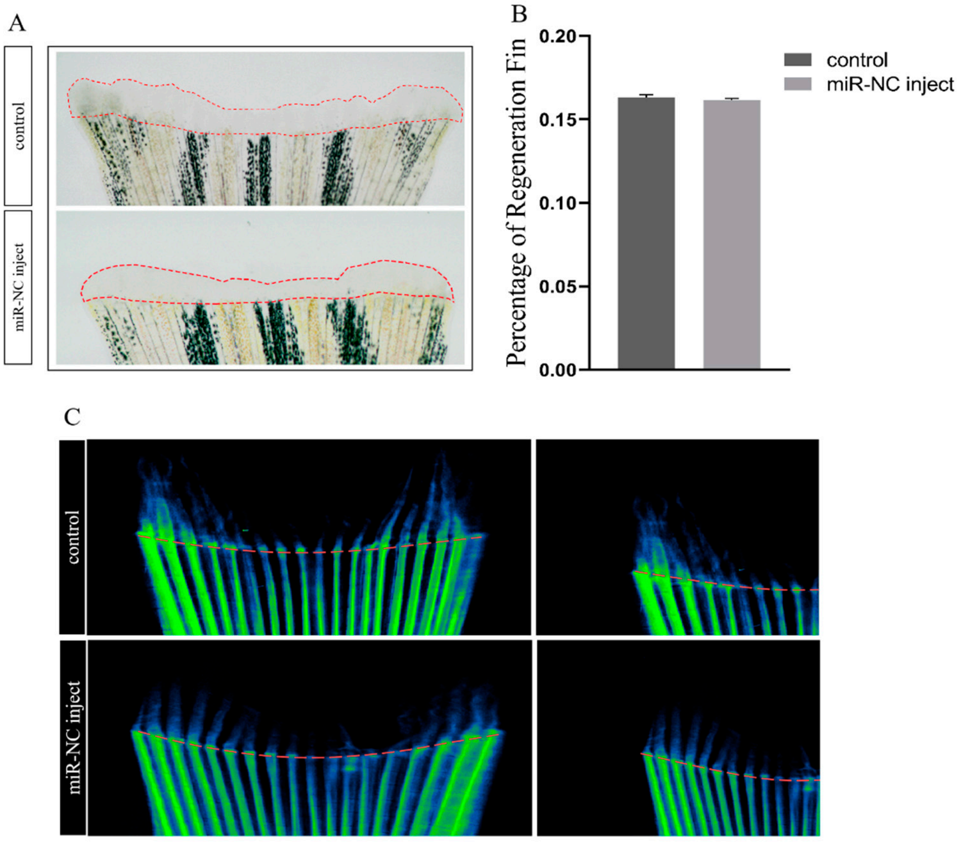

Figure S3. Analysis of phenotypic changes in caudal fin regeneration following miRNAs injection. (**A**) Regenerative phenotype of the 4 dpa regeneration group and the miRNA-NC injection group. (**B**) Percentage of the total caudal fin area of each group (n=4 fish in each group). (**C**) CT presentation of 4dpa regeneration group and miRNA-NC injection group. Dashed lines indicate the amputation plane. Plot values represent means ±s.d. Significant differences ($p < 0.05$) between treatment and control groups are indicated by asterisks above the bars.
