# Supplementary material for: The Regulatory Role of miRNAs in Zebrafish Fin Regeneration
Source: Int J Mol Sci. 2024 Sep 30;25(19):10542. doi: 10.3390/ijms251910542 (PMC11477159; doi:10.3390/ijms251910542)
Supplement: Supplementary file 1 [file ijms-25-10542-s001.zip › Supplementary Tables S1 and S2.pdf]

Table S1. Primers used in RT-qPCR in the present study.

Primer	Sequence (5′-3′)
dkk1a-F	ACATCCCAGGAGAACCACAG
dkk1a-R	AAACTTGTCCCTCTGTCAGCA
dkk1b-F	TCCTAAAAGAGGGCCAGGTC
dkk1b-R	TCCCTCGACTCAAGTCTGCT
wls/wnt1-F	TAAGCCAGGTGAGTGAGGGTCA
wls/wnt1-R	TCAGCGCTTGACTGCTCATCTC
wnt3a-F	GATGCCCGCTCTGCTATGAATC
wnt3a-R	CCGATGTTTCTCAACCACCATTTC
bmp2a-F	CGTCCGGTTCTCTTCAGG
bmp2a-R	GTGTCCGTCTGTGGTCCAC
bmp2b-F	CTGAAAACGATGACCCGAAC
bmp2b-R	AACTGCTGCGTTGTTTTTCC
bmp4-F	AGCAGTGCCTTCAAAGGTTG
bmp4-R	CATGGGGAAACAGTCCATGT
*runx2*a-F	ACGGTAATGGCTGGAAATGA
*runx2*a-R	GTCCGTCCACTGTGACCTTT
*runx2*b-F	AGCTTCACCCTGACGATTACA
*runx2*b-R	CCAGTTCACTGAGACGGTCA
sp7-F	TCCAGACCTCCAGTGTTTCC
sp7-R	ATGGACATCCCACCAAGAAG
shha-F1	GTGCAGCGGATATACACGGA
shha-R1	CTGCTGGAGTTTTGGGGGAA
dre-mir-338-F	CGTCCAGCATCAGTGATTTTGTTG
dre-miR-145-5p	GCCGTCCAGTTTTCCCAGGAATCCC
dre-miR-218a	GCCGCTTGTGCTTGATCTAACCATGTG
dre-mir-216b-F	GCTAATCTCTGCAGGCAACTGTGA
dre-mir-375-F	TGTTCGTTCGGCTCGCGTTA
dre-mir-216a-F	GCTAATCTCAGCTGGCAACTGTGA
dre-mir-146a-F	CGGTGAGAACTGAATTCCATAGATGG
dre-miR-15b-5p-F	GCCTAGCAGCACATCATGGTTTGTA
dre-miR-193a-3p-F	GCAACTGGCCTACAAAGTCCCAGT
dre-miR-21-F	GCCGTAGCTTATCAGACTGGTGTTGGC
dre-miR-724-F	GCCGCAAAGGGAATTTGCGACTGTT
dre-miR-455-5p-F	GCCGTATGTGCCCTTGGACTACATCG
dre-miR-142a-3p-F	GCCCGCCTAGTGTTTCCTACTTTATGGA
dre-miR-152-F	GCCGGTCAGTGCATGACAGAACTTTGG
dre-miR-101a-F	GCCCGCCACAGTACTGTGATAACTGAAG
dre-miR-205-5p-F	GCTACTCCTTCATTCCACCGGAGTCTG
dre-miR-181b-5p-F	GCCGAACATTCATTGCTGTCGGTGGG

Table S2. Primers used in ISH in the present study.

Primer	Sequence (5′-3′)
ish-*sp7*-F	TAGACATGACGCATCCTTACG
ish-*sp7*-R	GGTTAAATCTCCAGCAGTCCAC
ish-*yap1*-F	CCCAAGCTTGGGCTTCCTGACGGGTGGGAAC
ish-*yap1*-R	CGGAATTCCGATTGGGCAGCAGTTCGGTAT
ish-runx2a-F	CCACGCCGAACTCCTTCAATC
ish-runx2a-R	TCAATATGGCCGCCACACGGA
ish-runx2b-F	AGATCATCGCCGATCACCCG
ish-runx2b-R	CAAGTCTGTCGAACCTGGAAGA
